# Knowledge, Attitudes and Application of Critical Nutrient Supplementation in Vegan Diets among Healthcare Professionals—Survey Results from a Medical Congress on Plant-Based Nutrition

**DOI:** 10.3390/foods11244033

**Published:** 2022-12-14

**Authors:** Michael Jeitler, Maximilian Andreas Storz, Nico Steckhan, Dorothea Matthiae, Justina Dressler, Etienne Hanslian, Daniela A. Koppold, Farid I. Kandil, Andreas Michalsen, Christian S. Kessler

**Affiliations:** 1 Institute of Social Medicine, Epidemiology and Health Economics, Charité—Universitätsmedizin Berlin, Corporate Member of Freie Universität Berlin and Humboldt-Universität zu Berlin, 10117 Berlin, Germany; 2 Department for Complementary and Integrative Medicine, Immanuel Hospital Berlin, 14109 Berlin, Germany; 3 Department of Internal Medicine II, Center for Complementary Medicine, Freiburg University Hospital, Faculty of Medicine, University of Freiburg, 79106 Freiburg, Germany; 4 Digital Health Center, Hasso Plattner Institute, University of Potsdam, 14482 Potsdam, Germany

**Keywords:** plant-based diet, vegan diet, vegetarian diet, vitamin B12, critical micronutrients, nutritional deficiencies

## Abstract

Plant-based diets are associated with numerous health benefits but also bear risks of micronutrient deficiencies if inadequately planned. The risk of nutrient deficiencies can be reliably reduced by supplementation but requires risk-awareness. We distributed a paper-and-pencil questionnaire to *n* = 902 healthcare professionals attending a congress on plant-based nutrition (VegMed 2018, Berlin). On the day of the survey (21 April 2018), *n* = 475 questionnaires were returned and analyzed descriptively. Of the *n* = 213 strict vegan participants, 2% (*n* = 5) took no supplements at all. All supplementing vegans reported taking vitamin B12. Almost three-quarters of vegans (73%, *n* = 152) took vitamin D, and 22% (*n* = 45) reported taking omega-3 fatty acids. Iron was supplemented by 13% (*n* = 28), iodine by 12% (*n* = 25), calcium by 11% (*n* = 22), zinc by 7% (*n* = 14), magnesium by 5% (*n* = 11), and selenium by 4% (*n* = 9). For 11%, a supplement other than vitamin B12 was subjectively most important. Nearly 50% had their vitamin B12 levels laboratory tested at least once a year; nearly one-quarter reported testing every two years, and another one-quarter rarely or never. Participants following a vegan diet were better informed about institutional recommendations of the German Nutrition Society and the Academy of Nutrition and Dietetics for vegan diets than participants following vegetarian or omnivorous diets. Vegan nutrition in pregnancy/lactation period and childhood was considered most appropriate by vegans. Despite a high awareness of potential health risks associated with vitamin B12 deficiency on a strict vegan diet and a comprehensive understanding of the official dietary recommendations of nutrition societies, use of supplements and performance of regular laboratory tests were only moderate among vegan healthcare professionals. Considering the paramount importance of adequate supplementation of critical nutrients to avoid nutrient deficiencies, scientific and public discourse should be further facilitated. Further investigation of the supplementation behavior of vegan health professionals could be of particular interest, as a possible correlation with the quality of their own nutrition counseling is not inconceivable.

## 1. Introduction

Interest in plant-based diets has soared in recent decades for environmental, ethical and health reasons [1]. Plant-based diets, including vegetarian and vegan diets, are more sustainable than omnivorous diets and have repeatedly been associated with benefits for human and planetary health [2]. Vegan diets in particular are gaining social importance, with there being 1.3 million vegans in Germany in 2016 [3]. In nutritional medicine, plant-based diets are increasingly used as preventive, adjuvant, and therapeutic measures, especially in the context of lifestyle-associated diseases such as cardiovascular disease, cancer, metabolic syndrome, and type 2 diabetes mellitus [4,5,6,7,8].

Comprehensive studies have shown that inadequately planned vegan diets may adversely affect nutrient supply: In addition to essential amino acids and fatty acids, vitamins (B12, B2, and D) and minerals (iodine, selenium, zinc, and calcium) are considered potentially critical nutrients [9,10]. Most of these substances are readily available in plant sources and can therefore be consumed in sufficient quantities on a balanced whole-food plant-based diet [11,12,13].

Vitamin B12, on the other hand, is, according to current knowledge, absent in almost all plant foods [14]. Whether the content and usability of vitamin B12 from some plant sources, such as algae, is sufficient for humans is still subject of controversial discussions [15,16,17]. Notably, an adequate supply of vitamin B12 can be ensured by additional intake of supplements [14,18]. Vitamin B12 deficiency can result in severe health consequences such as neurological symptoms, macrocytic anemia, and psychiatric disorders [19,20].

In its 2016 position paper (expanded in 2020), the German Nutrition Society (Deutsche Gesellschaft für Ernährung, DGE) expressed cautious views on plant-based nutrition [11]. In a nutshell, vegan diets are not recommended by the DGE for children and during pregnancy and lactation. Furthermore, the DGE recommends reliable supplementation of vitamin B12. In contrast, the U.S. Academy of Nutrition and Dietetics (AND), stated in 1993 that a plant-based diet is acceptable at all stages of life and emphasized its health-promoting effects [21]. The ADE recommends reliable sources of vitamin B12, such as fortified foods or dietary supplements. The vitamin B12 intake of vegan-fed infants and children should be determined.

The current scientific discourse on vitamin B12 supplementation in plant-based nutrition is characterized by controversies and insecurities. It is unknown to which extent vegans follow nutritional recommendations of nutrition societies. This may apply for both regular vitamin B12 testing as well as its adequate supplementation. Vegans’ awareness of current institutional dietary recommendations has rarely been investigated. The aim of this study was to assess knowledge and risk awareness regarding the supplementation of critical nutrients of healthcare professionals in vegan diets.

## 2. Materials and Methods

### 2.1. Survey Participants

We invited all physically present participants (*n* = 902) of the “VegMed 2018” conference (Europe’s largest interprofessional conference on plant-based nutrition for healthcare professionals in Berlin, Germany) on 21 April 2018 to partake in an anonymous paper–pencil survey after giving informed consent. The VegMed conference took place in Berlin from 20 to 22 April 2018, for the 5th time in total and, being the largest European scientific conference for plant-based nutrition, attracted attendees from countries all over the world. Previous publications from VegMed conferences included personality profiles of and empathy for vegetarians and vegans [22,23,24]. This study was approved by the ethics committee of Charité—Universitätsmedizin Berlin (12 February 2018, EA4/016/18) and registered at clinicaltrials.gov under NCT03542591.

### 2.2. Questionnaire and Data Collection

As a first step, the survey was designed based on a literature search in the PubMed medical database (for details see Supplementary Materials S1). Current knowledge and research gaps were identified based on the most recent publications in the field. Based on this, characteristics were defined, which were then operationalized. The questionnaire consisted of self-designed non-validated questions and covered the following areas:

- Socioeconomic parameters: gender, age, occupation, dietary pattern
- Supplementation among vegans: type of supplement(s), dosage and frequency
- Laboratory checks among vegans
- Knowledge: knowledge of current guideline recommendations (DGE and AND), knowledge of the risk of vitamin B12 deficiency, knowledge about the necessity of vitamin B12 supplementation
- Attitudes towards vegan nutrition during sensitive phases of life (pregnancy/lactation period and childhood)

Supplementary Materials S1 presents the questions and associated response scales of the questionnaire in detail. We distributed the questionnaire to all attending healthcare professionals, regardless of their diet. However, funnel questions were used to inquire about supplementation in participants indicating a strict vegan diet. The questionnaire was originally designed in German language and subsequently translated into English without any changes in content or design. The translation was reviewed by three independent native English speakers to guarantee congruence between the German and English versions.

Data collection was carried out on a voluntary basis and anonymously. To optimize the response rate and to provide information about ethical aspects of the survey, conference participants were informed about the aims and benefits of the study, as well as about its anonymity and voluntariness before the questionnaires were distributed. To avoid redundant counting or multiple participation, the survey was simultaneously conducted at a fixed time (11.00–11.15 a.m.) in three different lecture halls of the building where the conference was hosted. The questionnaires were returned at the exits after leaving the lecture halls.

### 2.3. Data Management and Statistical Analysis

Data from questionnaires was entered into Limesurvey 2.9. Ten percent of entered data was double-checked by a second person, yielding an error rate of 0.03%. Survey participants were categorized as vegans, vegetarians or omnivores based on their self-reported dietary patterns. To detect associations between influencing variables (dietary pattern, occupation, or age) and the target variables, we used Chi-square tests to test for independence. If significant, post hoc paired Fisher tests or Chi-square tests were used. If the target variable was continuous the Kruskal–Wallis test as a non-parametric pendant of the ANOVA was used to detect differences between groups, and multiple post hoc comparisons were then computed using Dunn tests. To correct for multiple comparisons, *p*-values were adjusted using the Benjamin–Hochberg method. All computations were done inside the statistical programming language R 4.0 using the following packages: cluster, psych, rcompanion, tidyverse.

## 3. Results

### 3.1. Participants

We analyzed a total of *n* = 475 questionnaires (*n* = 412 German and *n* = 63 English questionnaires, corresponding to a response rate of 53%). Eighty-one percent (*n* = 385) of respondents were women and 19% (*n* = 90) were men. Mean age was 36.75 ± 13.97 years, with a range between 18 and 72 years. More than one third of the participants were students of health-related subjects (36%; *n* = 169), followed by medical doctors (27%; *n* = 126) and nutritionists (7%; *n* = 34). Table 1 shows the occupational distribution of the remaining participants. More than half of the participants (58%; *n* = 275) reported consumption of a vegan diet (Table 2). Of these, 77% (*n* = 213) considered themselves to be strict vegans and 23% (*n* = 62) as predominantly vegan. Sixteen percent (*n* = 77) reported a vegetarian diet. The remaining participants reported an omnivorous diet (26%, *n* = 119). The average age of vegans was 34.76 ± 12.55 years, and thus slightly lower than that of vegetarians (39.25 ± 13.92 years).

### 3.2. Supplementation

#### 3.2.1. Supplementation Prevalence among Vegans

Of the 213 strict vegan participants, 2% (*n* = 5) took no supplements at all. The vast majority (98%) took at least one supplement, and approximately 50% (*n* = 106) took three or more supplements (Supplementary Material S2, Table S1). Vitamin B12 was perceived to be the most important supplement (89%). Approximately 11% considered another supplement to be their most important one. Ranked by frequency, these included vitamin D (7%), iron (2%), and vitamin B complex, vitamin C, magnesium, and omega-3 fatty acids (<1% each).

#### 3.2.2. Mode of Application, Dosage and Frequency of Supplement Intake among Vegans

Vitamin B12 was the most frequently taken supplement (Table 3). All 208 supplementing vegans reported taking vitamin B12.

Of these 208, 45% (*n* = 105) supplemented vitamin B12 orally, 35% (*n* = 81) mucosally and 7% (*n* = 17) subcutaneously. Seventeen percent (*n* = 36) chose different vitamin B12 supplements simultaneously. Almost three quarters of vegans (73%, *n* = 152) took vitamin D. In most cases, vitamin D was applied orally (44%, *n* = 67) or mucosally (34%, *n* = 51). Other forms of application were rarely chosen. Additionally, 22% (*n* = 45) reported taking omega-3 fatty acids, mostly orally (47%, *n* = 21) and mucosally (29%, *n* = 13). Iron was supplemented by 13% (*n* = 28), iodine by 12% (*n* = 25), calcium by 11% (*n* = 22), zinc by 7% (*n* = 14), magnesium by 5% (*n* = 11), and selenium by 4% (*n* = 9).

Regarding dosage and frequency of supplementation, heterogeneous combinations were reported. Selected dosages ranged from 3µg to 1000 µg, with intake frequencies from once daily to once per week. Overall, 33% did not specify the dosage of their oral vitamin B12 supplement used.

### 3.3. Laboratory Checks among Vegans

Nearly half of vegan participants (49%; *n* = 104) reported having their vitamin B12 levels checked at least once a year. Approximately one quarter (24%; *n* = 52) indicated every 2 years, 13% (*n* = 27) rarely, and 10% (*n* = 22) never (Supplementary Material S2, Table S2). There was no significant correlation between the frequency of laboratory controls and occupation (*p* = 0.17), gender (*p* = 0.18), or age quartiles (*p* = 0.4).

### 3.4. Knowledge

#### 3.4.1. Knowledge about DGE and AND Recommendations

**Figure 1:**
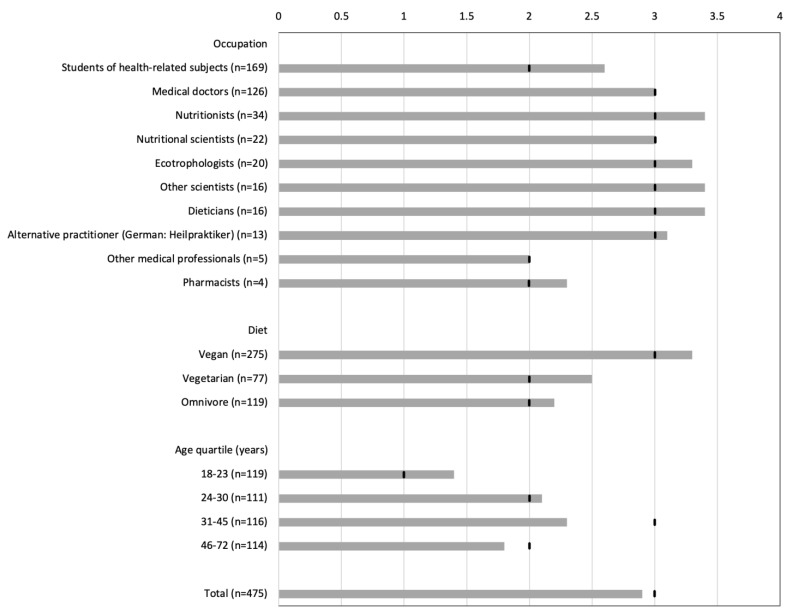
Score on knowledge of recommendations from nutrition societies (columns indicate mean values, vertical bars indicate median values).

The mean score of all survey participants was 2.9 out of 5 points, the median was 3 points (Figure 1), indicating a moderate knowledge of current guideline recommendations (DGE and AND). The youngest age group (18–24 years) showed a score of 1.4 points (median 1), and was thus well below the average. This age group was at least 60% comprised of students of the health sciences.

There was no statistical association between occupation and achieved score (*p* = 0.27), but there was a significant dependance between type of diet and achieved score (C* = 0.44, *p* < 0.001). Post hoc tests showed significant correlations for omnivore/vegan (*p* < 0.001) and vegetarian/vegan (*p* = 0.008). The test of the vegetarian/omnivore pair showed no correlation (*p* = 0.803). It could be assumed that vegan participants with an average score of 3.3 are better informed than participants with vegetarian (2.5) and omnivorous (2.2) diets.

There were differences between the age quartiles regarding the achieved score (*p* = 0.0015). Post hoc tests showed that there were differences between the youngest age quartile (18–23) and all older age quartiles: compared to 24–31 year olds (*p* = 0.002), 31–46 year olds (*p* = 0.0497) and 46–72 year olds (*p* = 0.0034). Comparisons between the other age groups revealed no significant differences.

#### 3.4.2. Knowledge of the Risk of Vitamin B12 Deficiency

The overall risk of vitamin B12 deficiency was rated “high” (median) among participants. Descriptive statistics revealed that 80% of vegans, 78% of omnivores, and 77% of vegetarians rated the risk of vitamin B12 deficiency as “high” or “very high”. Vegans were less likely to indicate “do not know” (0%) than omnivores (4%). The risk of vitamin B12 deficiency was rated as “very low” or “low” by 6% of vegan participants. Among vegetarians and omnivores, it was about 8% each.

We observed a significant association between the various types of practiced diets and the knowledge about the risk of vitamin B12 deficiency in a vegan diet (C* = 0.16, *p* = 0.038). Post hoc tests showed that after adjustment the knowledge about risk of vitamin B12 deficiency was significantly different between vegans and omnivores (*p* < 0.001). The comparison between vegetarians and vegans also showed a significant difference (*p* = 0.008). In contrast, we found no statistically significant difference between vegetarians and omnivores (*p* = 0.8). Knowledge level was dependent on neither occupation (*p* = 0.25) nor age quartiles (*p* = 0.68).

#### 3.4.3. Knowledge about the Necessity of Vitamin B12 Supplementation

Overall, vitamin B12 supplementation was considered “necessary” on average (median 1.5) among all participants. Ninety-two percent of vegans considered supplementation “necessary”, 6% “rather necessary”, 2% “rather not necessary” and 1% “not necessary”. Among vegetarians, 73% considered supplementation “necessary”, 21% “rather necessary” and 4% “not necessary”. In the omnivore group, only 64% considered vitamin B12 supplementation “necessary”. Statistical analysis revealed a strong association between dietary habits and knowledge of the need to supplement vitamin B12 (C* = 0.146, *p* < 0.001). Pairwise post hoc tests showed dependencies between vegans and vegetarians and between vegans and omnivores in terms of knowledge about the need to supplement vitamin B12 (*p* < 0.001 for each). There was no correlation between the vegetarian and omnivorous groups (*p* = 0.38) or between occupation (*p* = 0.895) or age quartiles in terms of knowledge (*p* = 0.32).

### 3.5. Attitudes towards Vegan Nutrition during Sensitive Phases of Life

#### 3.5.1. Pregnancy and Lactation Period

The statement that vegan nutrition is suitable during pregnancy and lactation period was “completely agreed” with (modal value) by vegans, “agreed” with (modal value) by vegetarians, and “disagreed” with (modal value) most frequently by omnivores (Supplementary Material S2, Table S3). There was an association between reported dietary habits and attitude towards vegan diet during pregnancy and lactation period (C* = 0.67, *p* < 0.001).

Post hoc tests showed that the attitude of vegans and omnivores (*p* < 0.001) and that of vegans and vegetarians (*p* < 0.001) differed. There was also a significant difference between omnivores and vegetarians (*p* = 0.008). There was no correlation with age quartiles (*p* = 0.25) or occupation (*p* = 0.4).

#### 3.5.2. Childhood

The statement that vegan nutrition is suitable during childhood was “totally agreed” with (modal value) by vegans, “agreed” with (modal value) by vegetarians, and “disagreed” with (modal value) most frequently by omnivores (Supplementary Material S2, Table S4).

There was a significant association between type of diet practiced and attitude towards vegan nutrition during childhood (C* = 0.70, *p* < 0.001). All pairwise post hoc comparisons of the three diets showed significant differences (*p* < 0.001). Calculated modal values showed a higher level of agreement among vegans compared to vegetarians and omnivores. There was no correlation between age quartiles (*p* = 0.10) or occupation (*p* = 0.34).

### 3.6. Perception of Nutrition Societies (DGE and AND)

Table 4 shows participants’ perception of professional societies. The competence of nutrition societies was most frequently or on average assessed as satisfactory (modal value and mean value: school grade 3).

Chi-square test for independence showed a correlation between the selected nutritional form and their perception of nutrition societies (C* = 0.361, *p* < 0.001). Post hoc tests showed that the evaluation of vegans did not differ significantly from that of vegetarians (*p* = 0.98) and between vegetarians and omnivores (*p* = 0.98). However, the perceptions of vegans and omnivores differed significantly (*p* < 0.001), with vegans (grade 3) rating nutrition societies worse than omnivores (grade 2).

There was also an initial correlation between occupation and perception (C* = 0.42, *p* = 0.006). However, post hoc comparisons between the individual occupational groups revealed no meaningful correlations as shown in Table 4.

There were no differences between age quartiles and perception of nutrition societies (*p* = 0.19).

## 4. Discussion

Western societies have observed an increasing interest in plant-based eating patterns in recent decades. Plant-based diets have emerged as a potent dietary strategy to reduce the risk from non-communicable chronic disease, including type 2 diabetes mellitus and cardiovascular diseases [8,25]. However, if inadequately planned, vegan diets, in particular, may also bear the risk of (micro-)nutrient deficiencies [26,27,28]. Data on health professionals’ knowledge about nutritional supplementation in plant-based diets in Germany is generally scarce, and little is known about its application in daily (clinical) practice. The present study sought to address this literature gap. We conducted a survey at Europe’s largest conference on plant-based nutrition (VegMed 2018 in Berlin, Germany) and surveyed *n* = 475 healthcare professionals. Of these, 213 reported strictly following a vegan diet.

Our data suggest that the vast majority of healthcare professionals following a strict vegan diet took supplements (98%), which is more than the general vegan population [10,29,30]. All supplementing vegan participants reported taking vitamin B12. Thus, the ratio of supplementers is far higher than in a 2003 study examining a vegan general population, where only 50% of participants reported B12 supplementation [10]. The ratio of B12 supplementing vegans in our study also exceeded the ratio of B12-supplemented vegan children in the VeChi Youth Study—traditionally regarded as a supplement-friendly cohort [31]. To the best of our knowledge, the supplementation rate in our study is so far unmatched in the available literature.

Overall, existing studies show that vegans are more likely to take supplements (46–80%) than the omnivorous general population (17–66%) [10,29,30]. The heterogeneity in the existing studies (as well as the low reproducibility) could be explained by the fact that most studies used different definitions of supplementation, form of application, dosage, and frequency. Nevertheless, the proportion of vegans in the general population not taking supplements appears high at 20–54% [10,29,30]. Thus, the significantly higher supplementation rate among vegan participants/healthcare professionals in our sample (as compared to vegan “laypeople”) is noteworthy.

The current DGE recommendations on vegan nutrition advise regular testing of the status of critical nutrients in blood or urine. Considering these recommendations, it is concerning that a substantial proportion of participants reported irregular laboratory checks, and that 11% of participants did not consider vitamin B12 to be the most important supplement. This appears to be of paramount importance, given that an even lower compliance with guidelines may be expected among “non-professionals” in the German general population.

Background knowledge and risk awareness are considered to influence compliance [32]. As reported by the German Federal Institute for Risk Assessment (Bundesinstitut für Risikobewertung, BfR), the general nutritional knowledge of vegans is at a high level due to their strong interest in nutritional topics [30]. Ninety-five percent of vegans are aware of the fact that a vegan diet can lead to vitamin B12 deficiency [30]. Vegan participants in our sample also demonstrated a high-risk awareness in terms of an insufficient vitamin B12 supplementation. In fact, their risk awareness was higher compared to that of other participants reporting a non-vegan diet. Furthermore, our data also suggest that vegan participants were best informed about institutional recommendations of professional societies.

In this context, it is also noteworthy to emphasize that the overall level of knowledge among the participants about recommendations for vegan nutrition by the DGE and AND was lower than expected based on their expertise. In particular, the youngest age group (18–23 years) appeared to be the least informed about the official recommendations of the DGE and AND. Since 60% of this group reported being students of health-related professions, the question arises as to what role statements by nutrition societies play in current curricula and education.

In addition, according to the BfR, institutions such as nutrition societies and trade associations are often viewed critically by vegans and are suspected of representing the omnivorous mainstream [30]. This might well apply to our sample, given that the competence of nutrition societies was only rated “satisfactory” (school grade: 3) by vegans. This is worse than the evaluation by omnivores (school grade: 2).

According to the report of the BfR, vegans distrust and reject the DGE with its risk emphasis and restraints towards vegan nutrition and regular recommendations of animal products (particularly dairy). For these reasons, vegans are said to rely on a very selective array of unofficial sources as the basis for their nutrition knowledge [30].

Apart from the fact that vegan nutrition during pregnancy, lactation period, and childhood raises ethical questions, the current state of studies on vegan nutrition in sensitive phases of life—viewed objectively—does not allow for an unequivocal statement in either a pro or contra direction.

The fact that vegans “completely agree[d]” (modal value) with vegan nutrition during pregnancy, lactation period, and childhood and favor this more than omnivores and vegetarians could mean that they trust the subjectively experienced positive effects of their diet, interpret the inadequate study situation correspondingly less objectively and perceive risks less critically. Then again, a high risk awareness and a clearly favorable attitude towards vegan nutrition seem to be able to coexist during sensitive phases of life.

Results of this study also emphasize the heterogeneity of possible supplementation methods of critical nutrients. On the one hand, this is a problem, because it complicates ‘broad-spectrum’ easy-to-practice supplementation. A clear ‘state of the art’ consensus on adequate supplementation, based on scientific clarity, could simplify the use of supplements for vegans, especially in the general population, as well as medical advice, consequently promoting adequate intake.

On the other hand, the variety of possible supplementation methods allow for a tailored and individual application based on specific needs. The complexity and individuality of the human metabolism and contradicting opinions on vitamin B12 assessment are barriers to the formulation of absolute statements for a broad public [33].

Our study has some important limitations. First, the study is not representative. The data were collected from participants of a plant-based nutrition conference, so the proportion of vegans and vegetarians is obviously different from that in the general population. Moreover, the congress was a professional event with the participation of experts, who are very likely familiar with nutrition science. In particular, there was a large difference in the participants’ sexes, as the study population consisted mostly of female students and female physicians. Overall, women, and especially female students, have more interest in PBD than men [34]. In a representative German survey conducted in 2021, twice as many 15 to 29 year olds followed a vegetarian or vegan diet as the general population. Most of them (70%) were female, 10.4% were vegetarians, and 2.3% vegans [34].

Second, we must acknowledge a potential selection bias due to the open and anonymous survey format and the extent to which the cohort is a representative subsample. Third, we had only a moderate response rate slightly exceeding the 50% threshold. Therefore, this study is rather exploratory in character. Fourth, self-assessment in written surveys always carries the potential for reporting bias, because false statements may be made due to social desirability or misinterpretation of the questions [35]. Reliability indicates how little or how strongly a test is distorted by measurement errors [35]. In written surveys, respondents’ reactive behavior may contribute to a reduction in reliability.

Fifth, the consistency of items used, and their underlying indicators, cannot be guaranteed regarding their function, and the measurement of one and the same characteristic. Validation in subsequent studies is thus warranted. For the topics “knowledge of critical nutrients in the vegan diet”, “official dietary recommendations”, “risk assessment of the vegan diet in sensitive life stages”, “performance of laboratory controls”, “supplementation of critical nutrients”, and “perceptions of professional societies”, indicators and items should be validated in the future.

Sixth, the fact that the survey was conducted on a voluntary basis may have reduced the overall objectivity. It can be assumed that volunteers are generally more interested in science, and more supportive of this particular research topic, and therefore more likely to calculate socially desirable responses. Said phenomenon could potentially reduce internal validity or representativeness. Finally, the fact that lectures on plant-based diets, nutrients, and their supplementation were given throughout the congress may have lowered objectivity and equivalence of the survey. Accordingly, at the time the written survey was administered, it is possible that some participants had already attended lectures or panel discussions on the topic, while other had not. However, the written survey took place at a fixed time for all congress participants under the same controlled conditions, which again favors measurement equivalence and objectivity. Acquiescence (tendency to say “yes”) was deliberately reduced by avoiding yes/no questions. Funnel questions and the answer option “do not know” were used as countermeasures to answers despite lack of knowledge, opinion, or relevance (non-attitudes).

Further investigations of vegans’ supplementation patterns and dietary recommendations may be of interest in the future. Overall, it may be of interest to examine the characteristics studied here for a different population, such as vegans in the general population. To explore reasons for inadequate supplementation, in-person interviews or recorded group discussions with vegans could be conducted as part of exploratory qualitative research before quantitative studies take place. For possible follow-up studies, this means asking which sources are used by vegans and which needs would have to be met in order to target the risk communication of an institution like the DGE.

The aim of future scientific studies in nutrition medicine should be to focus precisely on individuality in nutrition and metabolism. Regarding the supplementation of critical nutrients in vegan diets, this should mean that instead of a uniform “state of the art” recommendation, differentiated information about the advantages and disadvantages of individual supplements should be provided based on regular blood tests, in order to guarantee an individualized needs-based application.

Given the apparent need to accurately supplement critical nutrients on a vegan diet to avoid nutrient deficiencies, further scientific discourse should be facilitated. This seems even more necessary given that many of the participants are or will be involved in clinical patient care as medical professionals.

## 5. Conclusions

The risk of nutritional deficiencies can be reliably reduced by supplementation but requires risk-awareness. Manifest nutrient deficiencies, especially vitamin B12 deficiency, can have irreversible health effects. In view of the increased risk of nutrient deficiencies in unbalanced vegan diets, it should be a clear aim in the medical context to promote complianceof both expert and non-expert vegans in ensuring their nutrient status, to which the present data may contribute. As a primary exploration of a research gap, our data may serve as a call to action. It may be necessary, on the one hand, to improve the communication of appropriate dietary recommendations for a vegan diet and, on the other hand, to promote the implementation of these recommendations by (vegan) health professionals. Regarding the paramount importance of adequate supplementation of critical nutrients to avoid nutrient deficiencies in a vegan diet, further scientific and public discourses should be facilitated. In this context, the supplementation behavior of healthcare professionals who follow a vegetarian or vegan diet themselves may be of particular interest, as it can be assumed that correlations with the quality of their own counseling in the context of clinical patient care may exist.

## Figures and Tables

**Table 1:** Occupational distribution of the study population.

	Number (*n*)	Percentage
Students of health-related subjects	169	36%
Medical doctors	126	27%
Nutritionists	34	7%
Nutritional scientists	22	5%
Ecotrophologists	20	4%
Other scientists	16	3%
Dieticians	16	3%
Alternative practitioner (German: Heilpraktiker)	13	3%
Other medical professionals	5	1%
Pharmacists	4	1%
Not specified	50	10%
Total	475	100%

**Table 2:** Type of diet and age.

Diet	Number (*n*)	Percentage	Age (Years)		
			Mean	Standard Deviation	Median
Vegan	275	58%	34.76	12.55	30
Vegetarian	77	16%	39.25	13.92	40
Omnivore	119	26%	36.25	15.49	29
Not specified	4	<1%			
Total	475	100%	36.75	13.97	

**Table 3:** Application types of the most frequently mentioned supplements among vegans, in percent (*n* in parentheses).

	Total *n*	p.o.	muc.	s.c.	i.m.	f.f.	p.o.+ muc.	p.o.+ f.f.	p.o.+ i.m.	Other	Not Specified
Vitamin B12	235	44.7% (105)	34.5% (81)	7.2% (17)	2.1% (5)	1.3% (3)	2.1% (5)	1.3% (3)	0.1% (1)	3.8% (9)	2.6% (6)
Vitamin D	152	44.1% (67)	33.6% (51)	5.9% (9)	2.6% (4)	0.7% (1)	2.6% (4)	2% (3)	0.7% (1)	6.6% (10)	1.3% (2)
Omega-3	45	46.7% (21)	29% (13)	4.4% (2)	4.4% (2)	4.4% (2)	2.2% (1)	2.2% (1)	0	6.7% (3)	0
Iron	28	53.6% (15)	28.6% (8)	0	0	7.1% (2)	3.6% (1)	3.6% (1)	0	3.6% (1)	0
Iodine	25	48% (12)	12% (8)	0	0	4% (1)	0	4% (1)	0	12% (3)	0
Calcium	22	45.5% (10)	36.4 (8)	4.5% (1)	0	9.1% (2)	0	0	0	4.5% (1)	0
Zinc	14	50% (7)	28.6% (4)	7.1% (1)	7.1% (1)	0	0	7.1% (1)	0	0	0
Magnesium	11	45.6% (5)	27.3% (3)	9.1% (1)	0	0	0	0	0	18.2% (2)	0
Selenium	9	66.7% (6)	22.2% (2)	0	0	0	0	0	0	11.1% (1)	0
Total	541	45.8% (248)	32.9% (178)	5.7% (31)	2.2% (12)	2% (11)	2% (11)	1.8% (10)	0.4% (2)	5.5% (30)	1.5% (8)

p.o. = per os/oral; muc. = mucosal, via oral mucosa; s.c. = subcutaneous; i.m. = intramuscular; f.f. = fortified foods.

**Table 4:** Assessment of the competence of nutrition societies by means of school grades (1 = best to 5 = poor).

	*n*	Median
**Occupation**		
Students of health-related subjects	169	3
Medical doctors	126	3
Nutritionists	34	3
Nutritional scientists	22	2
Ecotrophologists	20	2
Other scientists	16	3
Dieticians	16	2
Alternative practitioner (German: Heilpraktiker)	13	3
Other medical professionals	5	3
Pharmacists	4	2
Not specified	50	3
**Diet**		
Vegan	275	3
Vegetarian	77	3
Omnivore	119	2
Not specified	4	4
**Age**		
18–23	119	3
24–30	111	3
31–45	116	3
46–72	114	3
Not specified	15	2
Total	475	3

## Data Availability

Data from the study are available upon reasonable request.

## Supplementary Materials

The following supporting information can be downloaded at: https://www.mdpi.com/article/10.3390/foods11244033/s1, Supplementary Material S1: E-Methods; Supplement Material S2, Table S1: Prevalence of supplementation among vegans, Supplement Material S2, Table S2: Frequency of laboratory tests among vegans, Supplement Material S2, Table S3: Attitude towards vegan diet during pregnancy and lactation period, Supplement Table S4: Attitude towards vegan diet during childhood.
