# Supplementary material for: Knowledge, Attitudes and Application of Critical Nutrient Supplementation in Vegan Diets among Healthcare Professionals—Survey Results from a Medical Congress on Plant-Based Nutrition
Source: Foods. 2022 Dec 14;11(24):4033. doi: 10.3390/foods11244033 (PMC9777782; doi:10.3390/foods11244033)
Supplement: Supplementary file 1 [file foods-11-04033-s001.zip › Supplementary Material S1.pdf]

Supplementary Materials S1: E-Methods

The Supplementary Materials S1 present in detail the survey and associated response scales of the questionnaire. The aim was to assess knowledge and risk awareness to supplementation of critical nutrients of healthcare professionals in vegan diets.

1 Literature search

For this purpose, *Pubmed* was searched for the search terms documented in Table 1. No studies were excluded from the search based on publication date or study design. The search also yielded study results that included some of the search terms but were found to be contextually irrelevant (Table 1).

Table 1: Documentation of the literature search

Topic	Search path	Number of publications displayed in PubMed	Number of publications with usable content
**Supplementation crit. nutrients in vegan diet**	Vegan* AND supplementation	87	10
	Vegan* AND supplementation AND prevalence	15	1
	Vegan* AND supplement* AND application	3	2
	Vegan* AND supplement* AND dosage	67	8
	Vegan* AND supplement* AND frequency	42	6
**Knowledge of vegans regarding crit. nutrients in vegan diet**	Vegan* AND knowledge AND nutrient*	7	0
	Vegan* AND knowledge AND risk	14	0
	Vegan* AND knowledge AND guideline	3	0
	Vegan* AND knowledge AND recommendation	1	0
**Risk assessment by vegans regarding crit. nutrients in vegan diet**	Vegan* AND evaluation AND risk	17	2
	Vegan* AND perception AND risk	0	0
	Vegan* AND opinion	58	0
**Perception of professional societies**	Vegan* AND perception AND association	0	0
	Vegan* AND (competence OR expertise) AND association	0	0
	Vegan* AND (competence OR expertise) AND guideline	0	0

2 Socio-demographic characteristics

The sociodemographic characteristics of age, gender, occupation and diet were recorded by self-report on the questionnaire. The surveyed participants were grouped according to their characteristics of socio-demographic variables (age, gender, occupation and diet), see Table 2. A t-test was used to test whether there was a correlation between age and the type of diet indicated.

Table 2: Sociodemographic characteristics

Sociodemographic characteristic	Characteristics (groups)
**Age quartiles (years)**	18 - 23
	24 - 30
	31 - 45
	46 - 72
**Gender**	Male
	Female
**Diet**	Vegan (strictly/predominantly)
	Vegetarian
	Omnivore
**Occupation**	Students of health-related subjects
	Medical doctors
	Nutritionists
	Nutritional scientists
	Ecotrophologists
	Other scientists
	Dieticians
	Alternative practitioner (German: Heilpraktiker)
	Other medical professionals
	Pharmacists

3 Supplementation

To record the supplementation characteristics, the type of supplement, dosage, frequency of intake and method of application as well as the subjectively most important supplement were asked. The answers were mainly given as free text. To indicate the form of application, one or more of the answer options "oral/subcutaneous/muscular/mucosal/fortified food/other" could be selected. The evaluation was descriptive: most frequently mentioned combinations in supplementation as well as the subjectively most important supplement were determined by manual counting.

4 Compliance with laboratory checks of own vitamin B12 status

In their nutritional guidelines for vegan diets, nutrition societies recommend regular laboratory checks of blood concentrations of critical nutrients such as vitamins. The question shown in Table 2 was asked to operationalize the characteristic "Frequency of laboratory checks of own vitamin B12 status".

Table 3: Frequency of laboratory checks of own vitamin B12 status.

How often do you have yourself tested for vitamin B12 in the laboratory (e.g., by taking blood or urine samples)?					
1x/quarter	Semi-annual	Annual	Every 2 years	Rare	Never

5 Knowledge

For the operationalization of the characteristic "knowledge of critical nutrients and dietary recommendations in vegan diets", neither standardized indicators nor validated questionnaires were available. Therefore, the questions described here were developed. The "knowledge about nutrition recommendations of *DGE* and *AND*" were recorded by means of the following five questions (see Table 4).

Table 4: Knowledge about recommendations from nutrition societies

	Question	Correct answer
**1**	What is the position of the *German Nutrition Society (Deutsche Gesellschaft für Ernährung, DGE)* on vegan diets during pregnancy and lactation period?	Against this
**2**	What is the position of the *German Nutrition Society (Deutsche Gesellschaft für Ernährung, DGE)* on vegan diets for children?	Against this
**3**	What is the position of the *German Nutrition Society (Deutsche Gesellschaft für Ernährung, DGE)* on the possibility of ensuring an adequate supply of all necessary nutrients on a vegan diet (with good nutritional planning, without taking supplements)?	Against this
**4**	What is the position of the *US Academy of Nutrition and Dietetics (AND)* on vegan diets during pregnancy and lactation period?	For this
**5**	What is the position of the *US Academy of Nutrition and Dietetics (AND)* on vegan diets for children?	For this

To determine a score, all applicable answers (1 point each) were summed up. The highest possible score was therefore 5. The determined score of 0 to 5 was generously used as an interval scale to calculate a mean value. A *chi-square test* was used to determine whether there

was a relationship between occupational group or type of diet and score. Age quartiles were first compared using the *Kruskal-Wallis test* and then (post-hoc) pairwise using *Dunn's test.* The next indicator for the characteristic "knowledge" was the "assessment of the risk of vitamin B12 deficiency due to vegan diet", see Table 5.

Table 5: Knowledge of the risk of vitamin B12 deficiency

How do you assess the risk of vitamin B12 deficiency on a vegan diet?					
Very low	Low	Moderate	High	Very high	Don't know

The knowledge about the need to supplement vitamin B12 was determined as follows (Table 6):

Table 6: Knowledge about the necessity of vitamin B12 supplementation

How do you assess the need for vitamin B12 supplementation in a vegan diet?				
Not necessary	Rather not necessary	Rather necessary	Necessary	Don't know

In order to detect possible correlations between influencing variables (occupation and type of diet) and the target variable (level of knowledge), the test for independence was first performed using a *chi-square test* followed by pairwise post-hoc comparisons (*chi-square test* or *Fisher test*). Whether differences exist between the age groups was tested using the *Kruskal-Wallis test* and then (post-hoc) using *Dunn's test.*

6 Attitude towards vegan nutrition in sensitive life phases

The variable "attitude towards vegan diet in sensitive life phases" refers to the risk of malnutrition with a vegan diet during life phases with special nutrient requirements such as pregnancy, lactation period, and childhood. The characteristic was assessed by the following two questions (Table 7):

Table 7: Attitude towards vegan diet in sensitive life phases

How do you feel about the following statements?				
**1 "Vegan diets are appropriate for women during pregnancy and lactation period."**				
Reject completely	Decline	Don't know	Agree	Totally agree

2 "Vegan diet is suitable for children."

Reject completely	Decline	Don't know	Agree	Totally agree

In order to detect correlations between influencing variables (occupation and type of diet) and the target variables, they were first tested for independence using a *chi-square test.* If significant, pairwise comparisons were then made using *Fisher's test* or *chi-square test*. The age quartiles were compared using *Kruskal-Wallis tests* and subsequent (post-hoc) *Dunn tests.*

7 Perception of nutrition societies

As a selective indicator for the perception of nutrition societies among vegans, the competence of nutrition societies DGE/AND was evaluated using a school grade scale from 1 (very good) to 5 (poor). The school grades given by the comparison groups were sorted descriptively using the calculated medians. To detect correlations between influencing variables (occupational and age group) and the outcome variables, testing for independence was first performed using a *chi-square test*. In the case of significance, post-hoc pairwise *Fisher test* or *chi-square test* was tested.

8 Statistical Analysis

First, the response behavior of each group (age, gender, diet, occupation) was presented descriptively, and the response of central tendency was presented in the form of median, modal value or arithmetic mean (depending on the scale level). The independent variables were mostly socio-demographic characteristics. The null hypothesis was defined as „there is no association between the variables". First, it was assessed whether there is a general dependency between two variables. If so, pairwise comparisons between possible combinations of characteristics (independent/dependent) were performed. If an association was shown, post-hoc pairwise comparisons of all combinations of characteristics with the dependent variable were performed in the case of more than two characteristic expressions of the independent variable. In conjunction with the descriptive statistics, any detected differences or correlations could be interpreted. The statistical tests used are described in the following:

For **target variables of a categorical nature,** dependence was first tested using a *chi-square test*, in which all values of the influencing variables and the target variables were included (group test). At points where a dependency between two variables was detected, the *corrected contingency coefficient C** with a value between 0 and 1 (the higher, the greater the dependency of two variables) was calculated. If the group test revealed a dependency of the variables, the

dependency was then tested in pairs (post-hoc) between two variable expressions. Each expression of an influence variable (e.g., occupational group) with its response behavior (target variable expressions) was compared in pairs with each other. The post-hoc tests were either the *Fisher test* (with less than 5 expected answers of the same expression) or the *chi-square test* (with at least 5 expected answers of the same expression). If all **variables** were **at least ordinal** (e.g. age quartiles), comparisons were first made using the *Kruskal-Wallis test.* The *Dunn test* then served as a post-hoc test. In all cases, if more than two pairwise comparisons were made, *Benjamini Hochberg* adjustment was performed to limit the *false discovery rate.* The statistical descriptive and analytical evaluation was performed using the program *R* 4.0.
