# Supplementary material for: Knowledge, Attitudes and Application of Critical Nutrient Supplementation in Vegan Diets among Healthcare Professionals—Survey Results from a Medical Congress on Plant-Based Nutrition
Source: Foods. 2022 Dec 14;11(24):4033. doi: 10.3390/foods11244033 (PMC9777782; doi:10.3390/foods11244033)
Supplement: Supplementary file 1 [file foods-11-04033-s001.zip › Supplementary Material S2.pdf]

Supplementary Material S2

Supplementary Material S2, Table S1: Prevalence of supplementation among vegans.

Number of supplements taken	*n*	%
0	5	2
1	32	15
2	70	33
3	51	24
4	31	15
5	24	11

Supplementary Material S2, Table S2: Frequency of laboratory tests among vegans.

	n	%
1x/quarter	3	1
Semiannual	25	12
Annual	76	36
Every 2 years	52	24
Rare	27	13
Never	22	10
Not specified	8	4

Supplementary Material S2, Table S3: Attitude towards vegan diet during pregnancy and lactation period.

	n	**Modal value**
**Occupation**		
Students of health-related subjects	169	Totally agree
Medical doctors	126	Totally agree
Nutritionists	34	Totally agree
Nutritional scientists	22	Agree
Ecotrophologists	20	Agree
Other scientists	16	Totally agree
Dieticians	16	Totally agree
Alternative practitioner (German: Heilpraktiker)	13	Totally agree
Other medical professionals	5	Totally agree
Pharmacists	4	Totally agree
Not specified	50	Agree
**Diet**		
Vegan	275	Totally agree
Vegetarian	77	Agree
Omnivor	119	Decline
Not specified	4	Don't know
**Age**		
18-23	119	Agree
24-30	111	Totally agree
31-45	116	Totally agree
46-72	114	Agree
Not specified	15	Totally agree

Supplement Table S4: Attitude towards vegan diet during childhood.

	n	**Modal value**
**Occupation**		
Students of health-related subjects	169	Totally agree
Medical doctors	126	Totally agree
Nutritionists	34	Totally agree
Nutritional scientists	22	Agree
Ecotrophologists	20	Agree
Other scientists	16	Totally agree
Dieticians	16	Totally agree
Alternative practitioner (German: Heilpraktiker)	13	Agree
Other medical professionals	5	Agree
Pharmacists	4	Totally agree
Not specified	50	Agree
**Diet**		
Vegan	275	Totally agree
Vegetarian	77	Agree
Omnivore	119	Decline
Not specified	4	Agree
**Age**		
18-23	119	Totally agree
24-30	111	Totally agree
31-45	116	Totally agree
46-72	114	Agree
Not specified	15	Totally agree
